# A review of once-a-day milking in dairy cow grazing systems*

**DOI:** 10.3168/jdsc.2022-0293

**Published:** 2023-02-09

**Authors:** N. Lopez-Villalobos, J.M.D.R. Jayawardana, L.R. McNaughton, R.E. Hickson

**Affiliations:** 1 School of Agriculture and Environment, Massey University, Palmerston North 4410, New Zealand; 2 Department of Animal Science, Faculty of Animal Science and Export Agriculture, Uva Wellassa University, Badulla 90000, Sri Lanka; 3 Livestock Improvement Corporation, Hamilton 3240, New Zealand; 4 Focus Genetics, 17C Mahia St., Ahuriri, Napier 4144, New Zealand

## Abstract

Twice-a-day (TAD) milking during the whole lactation is practiced in about 55% of New Zealand herds and once-a-day (OAD) milking during the whole lactation in about 10% of herds. The remainder of farmers use a mixture of TAD and OAD. Results from long-term comparisons show that over a full-lactation cows milked OAD, on average, had lower yields of milk (27%), fat (23%), and protein (24%) and higher percentages of fat and protein than cows milked TAD, but cows milked OAD for the entire lactation had better reproductive performance. Herds of cows milked OAD have higher mean 3-wk submission rate, 6-wk in-calf rate, and conception to the first service, and lower not-in-calf rate than the herds of cows milked TAD for the entire lactation. Farmers that have adopted OAD milking have culled cows that are unsuitable for OAD, and used sires selected on a OAD selection index to produce cow replacements that are more suitable for OAD milking. This OAD index includes the same traits that are included in the New Zealand national selection index with different relative economic weights on these traits plus including udder support, front teat placement, milking speed, and body capacity. A pasture-based milk production system based on OAD can be an alternative for many dairy farmers to maintain or increase farm profitability, with the additional benefits of better cow fertility and flexibility of using labor.

## Highlights

• Milking OAD during the whole lactation is practiced in about 10% of New Zealand herds.
• An OAD milking production system reduces milk production per cow, but improves cow fertility and quality of life for the farmer and farm staff.
• Cows suitable for OAD can be produced by sires selected on an OAD-selection index.

## Graphical Abstract

**Figure:**
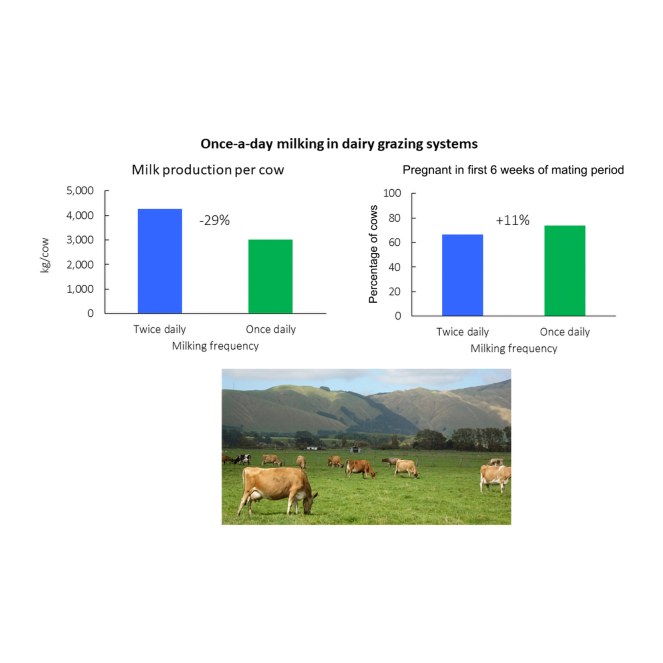
**Summary:** Once-a-day (OAD) milking during the whole lactation is practiced in about 10% of New Zealand herds, and there are also farmers milking OAD in Ireland and Australia. This study provided an updated review on OAD milking in dairy grazing systems. Cows milked OAD for the entire lactation had lower yields of milk, fat, and protein than cows milked twice per day, but cows milked OAD had better reproductive performance. The main reasons for farmers adopting OAD milking production systems are improvement of quality of life for the farmer and farm staff, reduction of farm costs, and improvement of cow fertility and animal welfare. Farmers can increase farm productivity and profitability by culling cows not suitable for OAD and producing heifer replacements from the best cows for OAD mated to bulls selected on an OAD-selection index.

Dairy farming in New Zealand is pasture based with most cows calving in spring. Twice-a-day (**TAD**) milking during the whole lactation is practiced in about 55% of New Zealand herds and once-a-day (**OAD**) during the whole lactation in about 10% of herds, with the remainder of the farmers using a mixture of TAD and OAD (Edwards, 2018a).

Davis et al. (1999) summarized short-term experiments comparing OAD and TAD and found that cows milked OAD produced, on average, 13% less milk than cows milked TAD. Rémond and Pomiès (2005) summarized experiments carried out in France and found that milk yield was reduced by 25% when cows were milked OAD for periods ranging from a few days to 2 to 3 mo, but milk losses and health hazards were higher when cows were milked OAD during the entire lactation period. Davis et al. (1998b) and Rémond and Pomiès (2005) identified individual differences in milk losses and suggested that there is an opportunity for selecting cows that are tolerant to OAD milking. Stelwagen et al. (2013) did a more extensive review on OAD miking considering short-, medium-, and long-term effects on OAD on milk production, milk composition and processability characteristics, energy balance, reproductive performance, and health and welfare of dairy cows. They reported that OAD milking reduces milk yield by approximately 22%, depending on stage of lactation, breed, and parity, and it may adversely affect lactation length and persistency. One conclusion of that study was that OAD milking fits well with more extensive dairy production systems, particularly those based on grazed pasture. The objective of the present study was to provide an updated review on OAD milking in dairy grazing systems, with focus on OAD as a production system; that is, milking cows OAD during the whole lactation and over multiple lactations.

A summary of results from studies evaluating the effect of OAD during the whole lactation is presented in Table 1. The experiment of Claesson et al. (1959) with twin cows in Sweden found that the average milk yield of cows milked OAD was reduced by about 50% in the first lactation and by about 40% in the second lactation in comparison with their twins milked TAD. A similar experiment carried out by Holmes et al. (1992) in New Zealand with few cows showed that cows milked OAD produced less milk, fat, protein, and lactose per cow than cows milked TAD. Rémond et al. (2004) reported that cows milked OAD produced less milk with higher concentrations of fat and protein with no differences in lactose concentration and SCC and were milked for 12 d fewer, compared with cows milked TAD. The trials conducted in New Zealand by Cooper and Clark (2001), Clark et al. (2006), and Dalley et al. (2008) found that cows milked OAD produced less milk (19 to 31%) and milk solids (fat plus protein) per cow (14 to 30%) than their TAD counterparts. Jersey (**J**) cows were less affected by OAD than Holstein-Friesian (**F**) and crossbred Holstein-Friesian × Jersey (**F × J**) cows. Milk per hectare was less for the OAD than TAD counterparts because the greater stocking rate for the OAD herds did not fully compensate for the milk loss per cow. Recent analyses of commercial herds by Lembeye et al. (2016) showed that F × J crossbred and J cows were less affected than F cows by OAD milking with a reduction in yield traits of 19.0%, whereas in F cows the reduction ranged between 19% to 25%. Jayawardana et al. (2022) reported levels of milk production for both TAD and OAD herds that were higher than those reported by Lembeye et al. (2016) and that cows in OAD herds produced less milk solids than cows in TAD herds. The reduction of milk production in OAD milking is hypothesized to be the result of a combination of acute and long-term alterations in mammary functions of OAD milking cows.

**Table 1:** Mean values for lactation yields of milk, fat, protein, lactose, and milk solids (kilograms of fat and protein) from dairy cows milked once a day (OAD) or twice a day (TAD) during the whole lactation

Study	Years of study	Breed^1^	Milking frequency	DIM	Milk (kg/cow)	Fat (kg/cow)	Protein (kg/cow)	Lactose (kg/cow)	Milk solids (kg/cow)	Milk solids (kg/ha)
Claesson et al. (1959)	1	SRW	TAD	280	3,525	140	121	182	261	
			OAD	280	1,792	73	64	90	137	
			Δ (%)		−49	−48	−47	−51	−47	
Holmes et al. (1992)	1		TAD	253	4,320	208	162	217	370	
			OAD	251	2,810	144	110	136	254	
			Δ (%)	−1	−35	−31	−32	−37	−31	
Cooper and Clark (2001)	1	F × J	TAD	269	3,543	177	131	171	307	922
			OAD	255	2,427	122	93	115	215	753
			Δ (%)	−5	−31	−31	−29	−33	−30	−18
Rémond et al. (2004)	1	Holstein	TAD	305	7,323	315	225	342	540	
			OAD	293	5,114	236	167	234	403	
			Δ (%)	−4	−30	−25	−26	−32	−25	
Clark et al. (2006)	4	F	TAD	244	4,234	187	149	205	336	1,051
			OAD	230	2,914	131	106	138	237	879
			Δ (%)	−6	−31	−30	−29	−33	−29	−16
		J	TAD	242	2,839	162	116	141	278	1,045
			OAD	229	2,211	129	94	107	222	979
			Δ (%)	−5	−22	−20	−19	−24	−20	−6
Dalley et al. (2008)	3	J	TAD/OAD	274	3,196	189	136		325	1,277
			OAD	269	2,592	163	117		280	1,219
			Δ (%)	−2	−19	−14	−14		−14	−5
O'Brien et al. (2009)	1	F	TAD	275	6,013	240	198	274	438	
			OAD	275	4,437	195	157	201	352	
			Δ (%)		−26	−19	−21	−27	−20	
Lembeye et al. (2016)	5	F	TAD		3,824	167	137		304	
			OAD		2,879	136	109		244	
			Δ (%)		−25	−19	−20		−20	
		F × J	TAD		3,446	169	131		300	
			OAD		2,787	143	110		254	
			Δ (%)		−19	−15	−16		−15	
		J	TAD		2,929	162	119		281	
			OAD		2,427	137	101		239	
			Δ (%)		−17	−15	−15		−15	
Jayawardana et al. (2022)	1	F, F × J, and J	TAD		4,860	231	186	243	417	
			OAD		3,201	169	131	158	300	
			Δ (%)		−34	−27	−30	−35	−28	

1 SRW = Swedish Red and White; F = Holstein-Friesian; J = Jersey and F × J = crossbred Holstein-Friesian × Jersey.

Research on the effect of OAD milking on cow fertility has been limited. Few studies have reported differences of reproductive performance between cows milked OAD and TAD during the entire lactation (Table 2). Clark et al. (2006) reported that cows milked OAD conceived 3 d earlier and took 5 d less from calving to conception than cows milked TAD. Compared with herds milked TAD, herds milked OAD had a higher (7.3%) percentage of cows inseminated in the first 21 d from the start of mating (**SR21**), and a higher percentage of cows that conceived in the first 21 d from the start of mating (**PR21**). Edwards (2018a) used data from commercial herds and found that percentages of cows that calved in the first 3 wk (**CR21**) and 6 wk (**CR42**) of the subsequent calving season were 5.0% higher in herds milked OAD compared with herds milked TAD. Hemming et al. (2018) also found that herds milked OAD had better fertility performance; a 7.7% higher mean SR21, 7.9% higher mean conception to first service, 10.4% higher mean PR42, 4.8% overall pregnancy rate, and 4.3% higher CR42 than TAD milking herds. The same trends were reported by Jayawardana et al. (2022) and they reported that the intervals from start of mating to first service and start of mating to conception were significantly shorter in cows milked OAD compared with TAD.

**Table 2:** Mean values for reproductive performance traits^1^ from dairy cows milked once a day (OAD) or twice a day (TAD) during the whole lactation

Study	Years of study	Breed^2^	Milking frequency	SMCO	SR21	PR21	PRFS	PR42	OPR	CR21	CR42
Clark et al. (2006)	4	F	TAD	28.2	79.3	37.4		67.3			
			OAD	26.1	89.7	41.8		69.8			
			Δ (%)	−7	13	12		4			
		J	TAD	28.4	90.1	39.3		67.6			
			OAD	23.8	94.4	50.4		75.1			
			Δ (%)	−16	5	28		11			
O'Brien et al. (2009)	1	F	TAD		63		50		73		
			OAD		73		40		90		
			Δ (%)		16		−20		23		
Edwards (2018a)	8	F, F × J, and J	TAD							59	82
			OAD							64	87
			Δ (%)							8	6
Hemming et al. (2018)	3	F, F × J, and J	TAD		76.9			64.4	82.4		83.9
			OAD		84.6			74.8	87.2		88.2
			Δ (%)		10			16	6		5
Jayawardana et al. (2022)	1	F, F × J, and J	TAD	27	77.8	46.4	54.1	67.4	85.8	59	82.4
			OAD	21.9	83.8	56.3	62.8	75.9	90.1	65.6	87.7
			Δ (%)	−19	8	21	16	13	5	11	6

1 SMCO = start of mating to conception (d); SR21 = cows inseminated in the first 21 d from the start of mating; PR21 = cows conceived in the first 21 d from the start of mating; PR42 = cows conceived in the first 42 d from the start of mating; PRFS = cows that conceived to their first service; OPR = overall pregnancy rate at end of the breeding season; CR21 = cows that calved in the first 21 d from the planned start of the calving; CR42 = cows that calved in the first 42 d from the planned start of the calving.

2 F = Holstein-Friesian; J = Jersey; and F × J = crossbred Holstein-Friesian × Jersey.

The overall results indicate that cows milked OAD have better reproductive performance than cows milked TAD. Cows milked OAD are inseminated earlier, become pregnant sooner in the mating season, and calve earlier in the next season than cows milked TAD. The reduced negative energy balance during the early lactation is hypothesized to be the reason for the improved reproductive performances of OAD milking cows than the TAD milking cows (Holmes et al., 1992).

A limited number of studies have reported the long-term effect of OAD milking on incidence of metabolic disorders, clinical mastitis, and lameness. O'Driscoll et al. (2010) evaluated the effect of milking frequency (OAD vs. TAD) at 2 nutritional levels on hoof health, locomotion, and lying behavior of cows. Cows milked OAD had lower sole lesion and white line disease scores, but higher heel erosion scores than cows milked TAD. There was an interaction between stage of lactation and milking frequency; cows milked OAD had higher overall locomotion scores than TAD cows, but this was reversed later in lactation. The conclusion of this study was that in general OAD milking resulted in improvements to hoof health and locomotion ability.

Long-term effects of OAD milking on SCC and clinical mastitis were reported in some studies (Holmes et al., 1992; Lacy-Hulbert et al., 2005; O'Brien et al., 2009). In the study by Holmes et al. (1992) with only 12 cows per milking frequency, SCC were significantly lower for the TAD milking group throughout lactation, even though there appeared to be no difference in the incidence of infection measured at the end of lactation. Lacy-Hulbert et al. (2005) reported the effect of OAD milking for complete lactations on mastitis and milk quality. Prevalence of IMI was not significantly or consistently higher for cows milked OAD compared with TAD. The SCC for uninfected cows milked OAD was approximately double that for TAD milked cows, although this difference was only significant after the first 1 to 2 mo of lactation had elapsed. Cows with minor or major pathogen infections also showed an approximate doubling of the SCC if milked OAD. The conclusion of this 4-yr study was that milking OAD consistently increases the individual cow SCC but does not significantly increase prevalence of clinical mastitis. Lembeye et al. (2016) analyzed herd-test records from commercial herds and confirmed no significant differences for SCS between herds that have adopted OAD and TAD milking for the entire lactation. It seems that once the cows and farmers adapt into OAD milking under grazing conditions with low levels of production, udder health is not affected.

Information about greenhouse gas (**GHG**) emissions in OAD milking systems is scarce, and this area needs further research. Greenhouse gas emissions can be evaluated in 2 ways, either looking at the total emissions of a farm or looking at the emissions per unit of product produced (efficiency). The impact of OAD milking on greenhouse gas emissions depends on which metric is used. In terms of efficiency per unit of product OAD milking would be expected to lead to higher emissions per unit of product, due to the negative impact of OAD milking on milk production. The impact of OAD milking production systems on total farm GHG emissions was evaluated by the Biological Emissions Reference Group (Reisinger et al., 2018) as a strategy to reduce biological GHG emissions. The Biological Emissions Reference Group concluded that from a climate change perspective, a OAD milking production system would reduce GHG emissions of the farm by 5% only to the extent that animals consume less feed, due to lower overall milk production. However, a life cycle assessment is required to comprehensively assess the full impact of OAD compared with TAD milking systems on carbon footprint at a farm and product level. The Biological Emissions Reference Group (Reisinger et al., 2018) indicated that apart from reduced feed demand as a result of lower milk production, OAD also has other impacts on the environment, for example, electricity savings from one less milking per day, reduced cowshed effluent, less water used for wash-down, less vehicle running for bringing the cows in, and fewer tanker loads of milk transported on the road. However, milking cows OAD means the cows will spend longer in the paddock, resulting in urine being deposited directly on pasture, which may limit the indirect emission benefits and reduces options to manage manure emissions and nitrate leaching. There are also complex interactions between genetic merit of the cows, stocking rate, reproductive performance of the herd, and cow longevity that affect milk solids production per cow and hectare with consequent effects on productivity, profitability, and GHG emissions of the production system. These need to be considered in a lifecycle analysis.

Few studies have reported on farm profitability of OAD milking compared with TAD milking systems. O'Brien et al. (2009) performed an economic evaluation of TAD and OAD milking using data generated from a trial comparing OAD and TAD at 2 nutritional levels. The stocking rates of the OAD groups were increased to produce the same amount of fat supplied by the farm under the milk quota system imposed by the European Union at that time. The results showed that farm profit was reduced for OAD compared with TAD milking by 24% and 59% at the high and low nutritional levels, respectively. This reduction was caused by reductions in milk revenue and increases in total costs. The same authors performed simulation scenarios and concluded that OAD will only suit the goals of some dairy farmers, creating flexibility on the use and requirement for labor.

Edwards (2018a) analyzed commercial herds that have adopted full-lactation OAD. On average, there was an 11% decrease in total farm milk solids production, but after 3 yr of OAD milking, production reached previous TAD production. The decrease in total farm milk solids production was influenced by the pre-OAD level of milk solid production (kg of milk solids per cow), with a smaller effect of OAD in herds producing ≤300 kg of milk solids per cow and a greater effect in herds producing 351 to 400 kg of milk solids per cow. Further economic analysis (Edwards, 2018b) reported average milk solids production per hectare decreased by 13% after adopting OAD; however, farm working expenses per hectare did not decrease, resulting in a decrease in proﬁtability per hectare. Further analysis of a subset of 33 OAD herds, with at least one season of pre-OAD data, grouped into quartiles on the basis of their pre-OAD labor efﬁciency, indicated that operating proﬁt per hectare of quartile 1 (least efﬁcient) increased by 23% after adopting OAD, with quartile 2, 3, and 4 decreasing by 1%, 10%, and 32%, respectively. Quartile 1 herds had the largest increase in cows per full-time equivalent, highlighting the importance of labor efﬁciency and cost reduction to proﬁt when milking OAD. The author indicated that proﬁtability can be maintained or increased after adopting OAD. In agreement with O'Brien et al. (2009) and Edwards (2018a,b), the decision to change from TAD to OAD milking requires a calculation of the trade-off between economic and lifestyle goals. The balance of the economic factors associated with OAD milking, such as lower milk production, labor requirement, potentially lower health costs associated with improvement on lameness and fertility, and better use of capital investments on the farm, must be set against the increased flexibility and time saving that may be achieved with OAD milking.

Dairy farming is a labor-intensive industry, with the milking process taking 43–58% of a standard 40-h week on TAD farms (Edwards et al., 2020). Milking OAD allows farmers to significantly reduce time spent in the cowshed (18–35% of a standard 40-h week). Farmers cite several benefits from changing to OAD milking. These include ease of attracting labor, reducing labor costs, an alternative option when feed shortfalls occur, herd expansion, allowing time to build capital, utilizing farmland with hilly terrain or long walking distances, and herd health and management (Bewsell et al., 2008). The number of farmers practicing OAD milking as system was about 200 in Ireland in 2021 (Teagasc, 2021) and 631 in New Zealand in 2015 (Edwards, 2018a). However, the evidence provided by dairy farmers that have adopted grazing systems in New Zealand, Ireland, and Australia supports the statement that “OAD is not for everyone, but it is an option” (Millerick and Millerick, 2020; p. 15). Each farm system has unique factors that may increase or decrease that individual farm's suitability to OAD milking.

All the experiments that have evaluated the effect of whole lactation OAD (Claesson et al., 1959; Holmes et al., 1992; Rémond et al., 2004; Clark et al., 2006; O'Brien et al., 2009) and the data set analyzed by Lembeye et al. (2016) show individual variation in the response to OAD milking. This indicates that there are genetic opportunities to select more suitable cows for OAD milking. Similarly, the differences observed among breeds in OAD performance relative to that of TAD performance are indicative of a genetic basis for the variation in the OAD yield response (Clark et al., 2006; Lembeye et al., 2016).

On a physiological basis, Stelwagen (2001) proposed that milk yield is a function of the number of secretory cells present in the udder and the metabolic activity of these cells. Both of these processes, alone or in combination, determine the milk yield potential of the mammary gland. Davis et al. (1998b) and Stelwagen (2001) concluded the short-term (i.e., weeks) effects of OAD on milk secretion are mediated via an up- or downregulation of cellular activity, whereas the long-term (i.e., months) effects are more likely related to changes in cell number. Davis et al. (1998a) proposed the first insight on the anatomical differences in the mammary gland between cows that are more or less tolerant of OAD milking. They suggested that the ability of the alveolar compartment to drain into the cistern is an important parameter and offers a possible explanation for the observation that cows that store a smaller proportion of their milk in the mammary cisternal compartment have greater milk losses when milked OAD.

Livestock Improvement Corporation (LIC) introduced a OAD index in 2003 for the genetic selection of cows and bulls as parents of cow replacements more suitable for OAD milking (McPherson et al., 2007). The OAD selection index was reviewed in 2019 (LIC, 2019). This new OAD selection index includes the same traits that are in the national selection index, with different relative emphasis, plus udder support, front teat placement, milking speed, and body capacity (Table 3).

**Table 3:** Relative emphasis of traits included in the national selection index (breeding worth) and the selection index for once-a-day milking (OAD SI) in New Zealand dairy cattle

Trait	Breeding worth	OAD SI
Milk	12	11
Fat	26	24
Protein	17	20
Live weight	12	10
Fertility	14	5
SCS	6	8
Survival	8	
BCS		
Milking speed		5
Capacity		4
Udder support		11
Front teat placement		2

Once-a-day milking has been adopted as a milk production system by a small proportion of farmers in Ireland, Australia, and New Zealand. A much higher proportion of farmers use OAD milking for a portion of the milking season. Reduction in milk production per cow is variable and depends on level of production of the herd (feeding level), breed, and individual variation, and some cows are only slightly affected. Individual variation in adaptability to OAD milking provides the opportunity to breed more suitable cows. There is now good evidence that herds that milk OAD have superior reproductive performance than herds milked TAD. Some farmers claim that by reducing operating costs and selecting the right cow for OAD, farm proﬁtability can be maintained or increased after adopting OAD. Better herd fertility can create an opportunity to reduce replacement rate, reduce replacement costs, and increase rate of genetic gain of the herd. The beneﬁts of OAD on the lifestyle and health of farmers needs to be considered in the economic evaluation of OAD production systems.

## References

[bib1] Bewsell D., Clark D.A., Dalley D.E. (2008). Understanding motivations to adopt once-a-day milking amongst New Zealand dairy farmers. J. Agric. Educ. Ext..

[bib2] Claesson O., Hansson A., Gustafsson N., Brännäng E. (1959). Studies on monozygous cattle twins. XVII. Once-a-day milking compared with twice-a-day milking. Acta Agriculturae Scandinavica.

[bib3] Clark D.A., Phyn C.V., Tong M.J., Collis S.J., Dalley D.E. (2006). A systems comparison of once-versus twice-daily milking of pastured dairy cows. J. Dairy Sci..

[bib4] Cooper C.V., Clark D.A. (2001). Once-a-day milking systems to improve productivity. Aust. J. Dairy Technol..

[bib5] Dalley D.E., Clough J.W., Hofman L.A., Phyn C.V.C., Clark D.A. (2008). Jersey cows milked once-a-day can produce 1200 kg milk solids per hectare. Proc. N.Z. Soc. Anim. Prod..

[bib6] Davis S.R., Farr V.C., Copeman P.J.A., Carruthers V.R., Knight C.H., Stelwagen K. (1998). Partitioning of milk accumulation between cisternal and alveolar compartments of the bovine udder: Relationship to production loss during once daily milking. J. Dairy Res..

[bib7] Davis S.R., Farr V.C., Stelwagen K. (1998). Once-daily milking of dairy cows: An appraisal. Proc. N.Z. Soc. Anim. Prod..

[bib8] Davis S.R., Farr V.C., Stelwagen K. (1999). Regulation of yield loss and milk composition during once-daily milking: A review. Livest. Prod. Sci..

[bib9] Edwards J.P. (2018). Comparison of milk production and herd characteristics in New Zealand herds milked once or twice a day. Anim. Prod. Sci..

[bib10] Edwards J.P. (2018). A comparison of profitability between farms that milk once or twice a day. Anim. Prod. Sci..

[bib11] Edwards J.P., Kuhn-Sherlock B., Dela Rue B.T., Eastwood C.R. (2020). Short communication: Technologies and milking practices that reduce hours of work and increase flexibility through milking efficiency in pasture-based dairy farm systems. J. Dairy Sci..

[bib12] Hemming N.V., McNaughton L.R., Couldrey C. (2018). Brief Communication: Reproductive performance of herds milked once a day all season compared with herds milked twice a day all season. N. Z. J. Anim. Sci. Prod..

[bib13] Holmes C.W., Wilson G.F., MacKenzie D.D.S., Purchas J. (1992). The effects of milking once daily throughout lactation on the performance of dairy cows grazing on pasture. Proc. N.Z. Soc. Anim. Prod..

[bib14] Jayawardana J.M.D.R., Lopez-Villalobos N., McNaughton L.R., Hickson R.E. (2022). Fertility of dairy cows milked once a day or twice a day in New Zealand. J. Dairy Sci..

[bib15] Lacy-Hulbert S.J., Dalley D.E., Clark D.A. (2005). The effects of once a day milking on mastitis and somatic cell count. Proc. N.Z. Soc. Anim. Prod..

[bib16] Lembeye F., Lopez-Villalobos N., Burke J.L., Davis S.R. (2016). Estimation of genetic parameters for milk traits in cows milked once- or twice-daily in New Zealand. Livest. Sci..

[bib17] LIC (2019). Once-a-day selection index. https://www.lic.co.nz/products-and-services/artificial-breeding/alpha/once-day-milking/.

[bib18] McPherson A.W., Pryce J.E., Winkelman A.M. (2007). The Once-a-Day Milking Conference, Hamilton, New Zealand.

[bib19] Millerick C., Millerick L. (2020). https://www.teagasc.ie/media/website/publications/2020/Catherine-Millerick-Once-a-Day-Milking-Conference-2020.pdf.

[bib20] O'Brien B., Gleeson D.E., Shalloo L. (2009). https://t-stor.teagasc.ie/bitstream/handle/11019/828/O%27Brien%2CB-EOPR_5521.pdf?sequence=1&isAllowed=y.

[bib21] O'Driscoll K., Gleeson D., O'Brien B., Boyle L. (2010). Effect of milking frequency and nutritional level on hoof health, locomotion score and lying behaviour of dairy cows. Livest. Sci..

[bib22] Reisinger A., Clark H., Abercrombie R., Aspin M., Ettema P., Harris M., Hoggard A., Newman M., Sneath G. (2018). Future options to reduce biological GHG emissions on-farm: Critical assumptions and national-scale impact. New Zealand Agricultural Greenhouse Gas Research Centre. Palmerston North. https://www.mpi.govt.nz/dmsdocument/32128/direct.

[bib23] Rémond B., Pomiès D. (2005). Once-daily milking of dairy cows: A review of recent French experiments. Anim. Res..

[bib24] Rémond B., Pomiès D., Dupont D., Chilliard Y. (2004). Once-a-day milking of multiparous Holstein cows throughout the entire lactation: Milk yield and composition, and nutritional status. Anim. Res..

[bib25] Stelwagen K. (2001). Effect of milking frequency on mammary functioning and shape of the lactation curve. J. Dairy Sci..

[bib26] Stelwagen K., Phyn C.V.C., Davis S.R., Guinard-Flament J., Pomiès D., Roche J.R., Kay J.K. (2013). Invited review: Reduced milking frequency: Milk production and management implications. J. Dairy Sci..

[bib27] Teagasc (2021). Once a Day Milking Conference 2021. https://www.teagasc.ie/news%E2%80%93events/national-events/events/oad-conference.php.

